# On measures of association among genetic variables

**DOI:** 10.1111/j.1365-2052.2012.02326.x

**Published:** 2012-06-28

**Authors:** Daniel Gianola, Eduardo Manfredi, Henner Simianer

**Affiliations:** * Department of Animal Sciences, University of Wisconsin-MadisonMadison, WI, 53706, USA; † Department of Animal and Aquacultural Sciences, Norwegian University of Life SciencesN-1432, Ås, Norway; ‡ Institut National de la Recherche Agronomique, UR631 Station d'AméliorationGénétique des Animaux, BP 52627, 32326, Castanet-Tolosan, France; § Department of Animal Sciences, Georg-August-Universität37075, Göttingen, Germany

**Keywords:** association indexes, entropy, gene frequencies, Kullback-Leibler distance, linkage disequilibrium, multivariate beta distributions, pleiotropy

## Abstract

Systems involving many variables are important in population and quantitative genetics, for example, in multi-trait prediction of breeding values and in exploration of multi-locus associations. We studied departures of the joint distribution of sets of genetic variables from independence. New measures of association based on notions of statistical distance between distributions are presented. These are more general than correlations, which are pairwise measures, and lack a clear interpretation beyond the bivariate normal distribution. Our measures are based on logarithmic (Kullback-Leibler) and on relative ‘distances’ between distributions. Indexes of association are developed and illustrated for quantitative genetics settings in which the joint distribution of the variables is either multivariate normal or multivariate-*t*, and we show how the indexes can be used to study linkage disequilibrium in a two-locus system with multiple alleles and present applications to systems of correlated beta distributions. Two multivariate beta and multivariate beta-binomial processes are examined, and new distributions are introduced: the GMS-Sarmanov multivariate beta and its beta-binomial counterpart.

## Introduction

The analysis of systems involving several variables is of great importance in population and quantitative genetics, for example, in multi-trait prediction of breeding values or in the study of networks using multi-locus data. The problem addressed here is that of measuring departures of the joint distribution of sets of genetic variables from stochastic independence. The target variables could be, for instance, pleiotropic additive effects of many loci under the infinitesimal model of Fisher (1918), correlated allelic frequencies of loci within bins of physical distance defined by numbers of base pairs, or sets of allelic frequencies that vary at random over clusters of individuals or sub-populations. Likewise, a study may involve systems of alleles whose joint distribution departs from independence, due either to proximity within a chromosome (linkage) or to forces such as random drift or selection favoring epistatic combinations, which may also create associations among loci on different chromosomes. When the problem is that of the study of the statistical association between allele states at different loci, this usually is referred to as gametic or ‘linkage disequilibrium’ (LD), with pairwise correlation measures typically employed for characterizing such association. If alleles at two loci are associated due to physical linkage, equilibrium (independence) is eventually attained asymptotically as generations of random mating accrue. LD has received enormous attention in population genetics (e.g., Hill 1974; Hedrick 1987; Lewontin 1988; McVean 2007) and has become an increasingly important topic of research in the light of emerging genomic data, e.g. single nucleotide polymorphism markers and sequence data enable the study of joint evolution of genomic blocks as well of as genome-wide association with complex disease or quantitative traits. This last area is one in which Professor Morris Soller has made important contributions, for example, a pioneering paper by Soller & Genizi (1978) in marker-assisted selection, and his 1990 work with Weller and Kashi on designs for inferring linkage between markers and quantitative trait loci in dairy cattle. The present paper is in his honor.

The objective of this work is to introduce new measures of association involving a system of variables based on notions of statistical distance between distributions. The resulting metrics are more general than correlations, which are pairwise measures only and lack a parametric interpretation beyond the bivariate normal distribution (e.g., Lewontin 1988), especially when associations are non-linear or when pairs of variables are neither independent nor identically distributed. For example, in the study of gametic disequilibrium, the distribution of correlation-based estimators is frequency dependent (Hedrick 1987), thus complicating comparisons among populations and meta-analyses. Also, there are situations such as in a multivariate *t-distribution* with a diagonal covariance matrix in which variables are uncorrelated and, yet, stochastically dependent. This illustrates that lack of correlation does not provide definitive evidence of independence: in this example, variable *X*, say, informs about *Y* even though uncorrelated. A related question is the inability of correlation to measure association between, say, 10 loci or 20 pleiotropic effects. The measures of association developed in this study are based on departure between the distributions of the variables under independence and association assumptions. Indexes measuring association between random variables, irrespective of their number or of the form of the joint distribution, are suggested and illustrated.

The article is organized as follows. Following this introduction, measures based on logarithmic and on relative ‘distances’ between distributions, as well as indexes of association, are presented. Subsequently, these indexes are developed and illustrated for quantitative genetics settings in which the joint distribution of the variables under consideration is either multivariate normal or multivariate-*t*. The fourth section shows how these indexes can be used to study LD in a two-locus system with multiple alleles; here, a correlation would not have much meaning, if at all. The fifth section presents applications to systems of correlated beta distributions; all these are generalizations of the univariate beta distribution, which has been used extensively in population genetics to study evolution of gene frequencies. In particular, two multivariate beta and multivariate beta-binomial processes are discussed, and new distributions are introduced: the GMS-Sarmanov multivariate beta and its beta-binomial counterpart. The paper concludes with a discussion of the concepts and of other procedures that have been proposed for study of multi-locus LD. Mathematical details are relegated to an appendix.

## Measuring association among variables

To motivate the approach used here, consider a pair *X*, *Y* of random variables with realized values *x*, *y*; the ideas generalize readily to distributions of higher dimension simply by replacing scalars by vectors. Let *p*(*x,y*) and *p*(*x*) × *p*(*y*) be the densities of the joint distributions under association and independence, respectively; *p*(*x*) and *p*(*y*) are the marginal densities of *X* and Y respectively. Association occurs whenever *p*(*y*|*x*) (or *p*(*x*|*y*)) differs from *p*(*y*) (or *p*(*x*)). Measures of dependence (association) can be derived from the density ratio

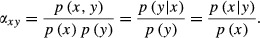
(1)

If *α*_*xy*_ = 1 for all pairs of values, the ‘distance’ between distributions is 0 and independence holds. Values of α_*xy*_ larger or smaller than 1 suggest statistical dependence. For example, *α*_*xy*_ larger than 1 indicates ‘coupling’, i.e. the density of certain pairs of values is higher than expected under independence; values smaller than 1 would be indicative of ‘repulsion’. Likewise, strong departures of log *α*_*xy*_ from 0 would be indicative of an association between *x* and *y*.

We discuss two measures of association based on either expected logarithmic distance between distributions or on expected relative distance, as conveyed by the average values of log *α*_*xy*_ and of *α*_*xy*_ respectively. These measures are used subsequently to develop indexes of association, irrespective of the number of variables in a system.

### Association based on Kullback-Leibler distance

The Kullback-Leibler (KL) logarithmic distance (Kullback 1968) is defined as the sum of two KL discrepancies between the distributions: one under association and the other under independence. The first discrepancy measures departure from association when the latter is true, and the other measures departure from independence when this attribute holds. The two discrepancies are positive by construction and are defined as

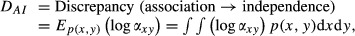

which measures discrepancy when it is true that *X* and *Y* are associated, and

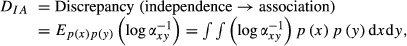

measuring discrepancy when independence holds; the range of integration depends on the random processes involved. If the two distributions are identical, both discrepancies are null; on the other hand, large discrepancies reflect stochastic dependence. Above, *E*_*p*(*x,y*)_ and *E*_*p*(*x*)*p*(*y*)_ denote expectations taken under the assumptions that (*X,Y*) follow either a bivariate distribution (association) or that these variables are independently distributed.

A connection with the likelihood ratio statistic is fairly direct. Suppose that the parameters of the distributions under independence and association are estimated by maximum likelihood (ML), and that these estimates are regarded as if they were ‘true’ values. Then, log *α*_*xy*_, with the densities evaluated at the corresponding ML estimates would be the log of a ratio between maximized likelihoods. In *D*_*AI*_ one would be taking expectations of the log-likelihood ratio over all possible realizations expected if the ‘full model’ (association) were true; on the other hand, in *D*_*IA*_ the expectations of the reciprocal of the log-likelihood would be taken under the assumption of independence. In this context, *D*_*AI*_ and *D*_*IA*_ are averages of log-likelihood ratios, as opposed to an evaluation at a single realization (in ML estimation parameters vary whereas observations are taken as fixed). Note that *D*_*AI*_ and *D*_*IA*_ do not involve any requirement of ‘nesting’ of models, contrary to likelihood ratio tests under regularity conditions, but this is a different issue.

The discrepancies are such that *D*_*AI*_ ≠ *D*_*IA*_, and a unique, symmetric KL distance is defined (Kullback 1968) as

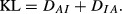
(2)

Then, an index of association (taking values between 0 and 1) is obtained by normalizing *D*_*IA*_ as

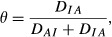
(3)
with

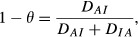

provided that KL is not null. A large value of *D*_*IA*_ relative to *D*_*AI*_ (or the opposite) is indicative of situations in which there is more association than what would be expected under independence so that *θ* or 1 − *θ* would be close to 1. Thus, values of *θ* or of 1 − *θ* away from 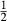 provide evidence of discrepancy between the hypotheses of random ensembles of (*x,y*) pairs vs. associated ensembles.

There is no closed form for *D*_*IA*_ or *D*_*AI*_ for many distributions, in which case these metrics must be calculated numerically or estimated using Monte Carlo methods, given some parameter values. If association is weak, the two logarithmic discrepancies and KL are nearly null, and this can lead to very unstable, even absurd, Monte Carlo estimates of *θ*. Also, the KL distance and the two discrepancies are logarithmic, and this perhaps provides a less intuitive notion of ‘distance’ than one based directly on *α*_*xy*_. An alternative ‘distance’ measure is presented immediately below.

### Association based on relative distance

Define the relative discrepancies

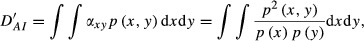
(4)

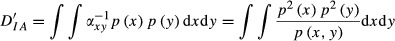
(5)
and

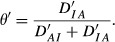
(6)

The metric 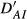 gives the expected value of *α*_*xy*_ under association and 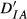 gives the expectation of 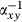 under the assumption of independence. These expectations are both positive, by construction, and values of *θ*^′^ away from 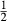 can be construed as reflecting departure from independence. For example, 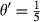 or 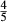 would suggest association. The connection with likelihood ratios surfaces again; for example, 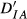 is the average likelihood-ratio over all possible realizations of the data under the independence assumption.

In what follows, the proposed indexes are examined for some distributions that are used often in quantitative and population genetics research.

## Multivariate Gaussian and *t*-distributed variables

In quantitative genetics, the analysis often focuses on a set of continuous correlated variables 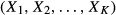 that follow some multivariate distribution; these variates could be, for example, additive genetic effects for *K* traits. Suppose that a matrix of estimates of additive genetic correlations is available (and taken as a ‘true’ matrix), and that we seek to arrive at a single measure of association among the *K* genetic values, as opposed to the 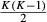 pairwise measures provided by the matrix of genetic correlations. Below, examples of multivariate normal distributions with *K* = 2, 3, 4 and of a bivariate *t*-distribution are considered to illustrate how *θ* behaves.

### Bivariate normal distribution

Let *X*_1_ and *X*_2_ be two standardized normally distributed genetic values with correlation *ρ*. For this distribution 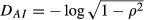 and 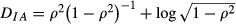 (e.g. Sorensen & Gianola 2002). When the correlation is null, the two discrepancies are 0; when the correlation is perfect, the discrepancies are both *∞*. Thus, KL = *ρ*^2^(1 − *ρ*^2^)^−1^, which is 0 when the two random variables are independent and *∞* under a perfect correlation. Then

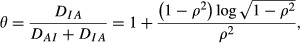
(7)
measures discrepancy away from the independence situation when the latter holds, relative to the KL distance between the two distributions. It can be shown that 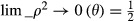 and 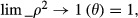 providing the lower and upper bounds of *θ* respectively.

**Figure 1:**
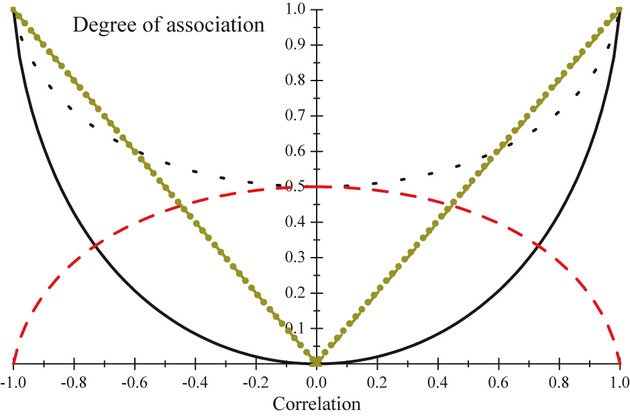
Measures of association of two bivariate Gaussian variables as a function of their correlation (ρ). The straight lines give the strength of the association as measured by the absolute value of ρ. The dotted (‘holds water’: θ) and dashed (‘spills water’: 1 − θ) lines depict the relative contributions to the Kullback-Leibler distance due to discrepancies under independence and dependence models, respectively. Values of the association measure γ=2θ − 1 are represented by the dark solid line.

The values of *θ* and of 1 − *θ* are plotted against ρ in Fig. 1. The dotted line (‘holds water’) gives the relative discrepancy (*θ*) away from the independence models when this is true, and the dashed line (‘spills water’) depicts the trajectory of 1 − *θ*, i.e. the relative discrepancy under association; note that a given curve is a mirror image of the other. As the absolute value of the correlation increases *θ* → 1, as one would expect. Since an association index with values ranging between 0 and 1 may be easier to interpret, an alternative measure could be

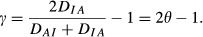


However, *γ* takes values in [0,1] provided that 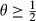, which holds for a bivariate Gaussian distribution, but not so in general. The relationship between *γ* and *ρ* for this Gaussian model is also shown in Fig. 1 (solid line), and the association is suggested as weaker when measured by *θ* than when assessed with the correlation *ρ*. For example, if 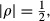 *θ* is smaller than 0.15. In multivariate systems or in other distributions, *θ* can be much smaller than 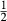 so that *γ* takes negative values, as illustrated below, so it turns out that *θ* often provides an easier to interpret measure of association than does *γ*.

### Multivariate normal distribution

The metrics under consideration can be applied to distributions with any number of dimensions, leading to measures of departure from independence in more general situations, such as when alleles in a multiple-locus system are studied. To illustrate, consider the *K*-dimensional multivariate Gaussian distribution (**x**∼*N*(**0,R**), where variates are in standard deviation units so that **R** is a correlation matrix; under independence (**x**∼*N*(**0,I**). It can be shown (Penny & Roberts 2002) that

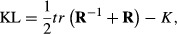
(8)

Where *tr*(.) denotes the sum of the diagonal elements of a squared matrix such as **R**. Further,

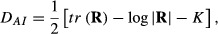
(9)
and

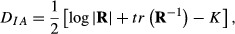
(10)
so that

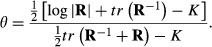
(11)

Examples of several multivariate normal distributions are given next.

#### Equi-correlated trivariate normal distribution

Let three variables be equi-correlated with correlation *ρ*, so that

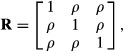

with 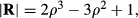 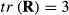 and 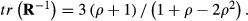 Using (8)-(11) yields

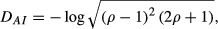


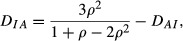


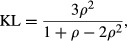

and

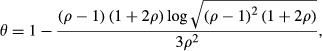
(12)

**Figure 2:**
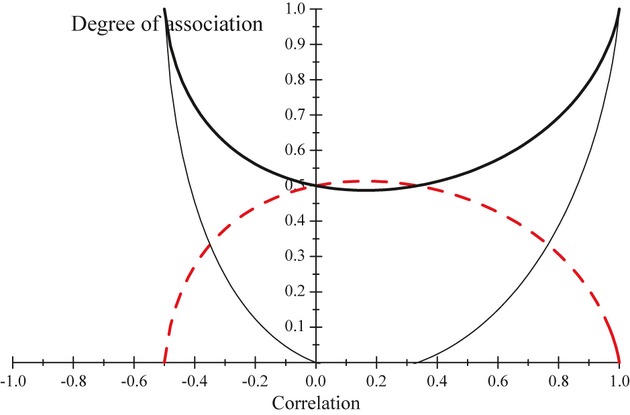
Indexes of association as a function of the coefficient of correlation in an equi-correlated trivariate normal distribution. The thick solid line gives the relative Kullback-Leibler discrepancy between distributions when independence holds (θ), and the dashed line gives the relative discrepancy when association is true (1 − θ). The thin line gives the trajectory of γ=2θ − 1.

With 0 < *θ* < 1. For example, for *ρ* = 0.25, 0.50 and 0.75, the index of association *θ* takes values 0.49, 0.54 and 0.66 respectively. Figure 2 depicts *θ*, 1 − *θ* and *θ* as a function of the coefficient of correlation. At *ρ* = 0, *θ* = 0.5, but then it decreases slightly as the correlation increases such that *γ* does not attain a positive value until *γ* reaches a value of about 0.3. Note that *θ* is not symmetric with respect to *ρ* (the same is true of |**R**|); for instance, when 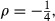 *θ* = 0.59 so that association would be viewed as stronger than when 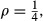 since *θ* = 0.49 then. This is a consequence of the values that the discrepancies *D*_*IA*_ and *D*_*AI*_ take at varying values of *ρ*.

#### Tetra-variate normal distributions

Alternative measures can be derived from eigenvalues of a correlation matrix; however, this is sensible only for distributions in which linear relationships between variables are meaningful. The index *θ*, derived from notions of statistical distance, does not have this limitation because KL discrepancies have a general meaning and can be calculated for any distribution, at least numerically, given values of the parameters. Naturally, any single measure of association in a multivariate distribution becomes less transparent as the system of variables increases in dimension.

Let *K* = 4 and suppose the variables are equi-correlated with coefficient *ρ*. Then, 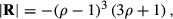 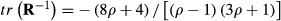 and

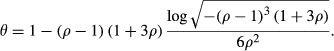
(13)

**Figure 3:**
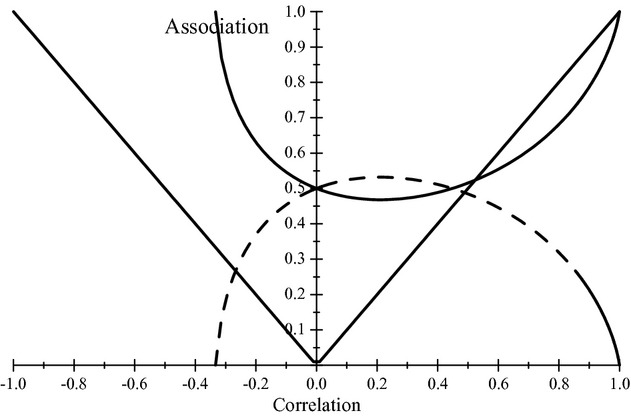
Discrepancies from independence to association (θ, solid line), and from association to independence (1 − θ, dashed line) as a fraction of the Kullback-Leibler distance between two tetravariate normal distributions. The straight lines give the absolute values of the correlation ρ.

Figure 3 displays how *θ* and 1 − *θ* vary against *ρ*. At *θ* = 0 one has 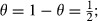 *θ* decreases somewhat subsequently and then increases, attaining 0.5 again at about *ρ* = 0.45. The values of the index of association suggest that the independence and association models are not ‘too distant’ unless the correlation is below −0.10, if negative, or above *ρ* = 0.50, if positive.

Let now *K* = 4 and take as correlation matrix

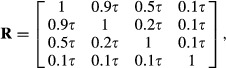

where 0 ≤ *τ* ≤ 1, so that when *t* = 0 the four variables are independent. For *t* = 1, log |**R**| = −2.5459 and *tr*(**R**^−1^) = 24.8980, yielding *θ* = 0.8782. Likewise, for 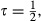 log |**R**| = −0.2960 and *tr*(**R**^−1^) = 4.6540, so that *θ* = 0.5473. This illustrates that the strength of the association is proportional to the strength of inter-correlation, and that *θ* provides a metric for measuring departure from independence.

We finish this section by posing the following question: what does *θ* say that is not informed by all possible pairwise correlations? Again, let *K* = 4 and take as correlation matrices

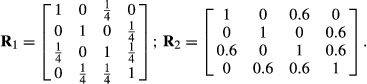


The salient point is that the correlation structure is such that while pairs of variables (1,3), (2,4) and (3,4) are correlated, (1,2) are independent.

What is the overall strength of the association in the system? Calculations yield *θ*_1_ = 0.54 and *θ*_2_ = 0.91 for the networks of variables characterized by 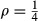 and 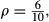 respectively. While the correlations measure association between pairs of nodes, *θ* gives an indication of the overall degree of association.

### Bivariate *t*-distribution

As mentioned, there are situations in which random variables are jointly dependent, but yet, their correlation is 0, which would incorrectly suggest that *X* does not inform about *Y*. This highlights limitations of the correlation parameter as a metric for statistical dependence between sets of variables. One such case is the multivariate-*t* distribution, in which variables can be statistically dependent, yet uncorrelated. The univariate and multivariate *t-distributions* appear in robust linear regression models for quantitative genetic analysis based on pedigrees (e.g., Strandén & Gianola 1998; Sorensen & Gianola 2002; Rosa *et al*. 2003). The univariate-*t* distribution has also been used as a prior in Bayesian linear regression models for genome-enabled prediction of single traits (Meuwissen *et al*. 2001; Gianola *et al*. 2009).

Suppose two variables, e.g. SNP effects on a pair of traits, follow a bivariate *t-*distribution with a null mean vector, *ν* degrees of freedom and a scale matrix that is equal to a 2 × 2 identity matrix; here, the two effects are uncorrelated but not independent, as shown in the Appendix. For this situation, the density ratio (1) is

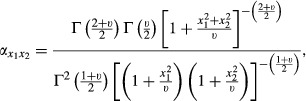

so that the relative discrepancies in (4) and (5) become

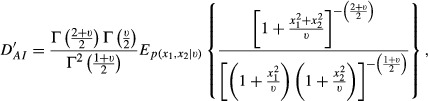

and

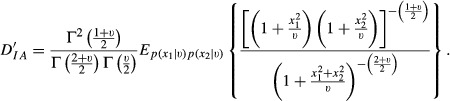


The expectations above do not have closed forms but can be evaluated using Monte Carlo methods: given the value of the degrees of freedom parameter, random numbers can be drawn from a bivariate *t*-distribution and from two independent univariate *t*-distributions, to approximate *D′*_*AI*_ *and D′*_*IA*_.

The index of association θ′ in (6) was computed for *t*-distributions with *μ* = 1, 2, 4, 6, 8, 20 and 100 000. The last value produces an approximation of the joint distribution of two independent normal variates, whereas the setting with *μ* = 1 evaluates distance between a ‘bivariate Cauchy’ distribution and the distribution of two identically and independently distributed Cauchy random variables. The *t-*processes with *μ* = 1, 2, 4 do not have a finite variance, but the distributions are proper (i.e., the density integrates to 1). As shown in Table 1, with sets of 1 million random numbers used for evaluating *D′*_*AI*_, *D′*_*IA*_ and *θ′*, the ‘distance’ *D′*_*AI*_ from association to independence decreases as *ν* increases, approaching 1, whereas the distance from independence to association *D′*_*IA*_ increases, approaching 1 as well. With *ν* = 100 000 the random variables are essentially independent since 
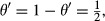
 leading to *γ′* = 0, the setting *ν* = 1 produces the strongest possible association, with *θ′* = 0 and both 1 − *θ′* and *θ*′ equal to 1.

**Table 1:** Departures of zero-mean bivariate *t-*distributions with a 2 × 2 identity matrix as scale parameter (so their correlation is null) from independence as measured by *D′*_*AI*_, *D′*_*IA*_ and by the indexes of association *θ′* = *D′*_*IA*_/(*D′*_*AI*_ + *D′*_*IA*_), 1 − *θ′* and *γ* ′ = 2(1 − *θ′*) − *1* (when 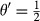 the random variables are independent)

Degrees of freedom	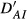	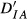	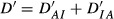	*θ*^′^	1 − *θ*^′^	*γ*^′^
1	7.28 × 10^9^	0.4376	7.28 × 10^9^	0.0000	1.0000	1.0000
2	63438837	0.6424	6348838	0.0000	1.0000	1.0000
4	20.7443	0.7947	21.5390	0.0369	0.9631	0.9262
6	2.6299	0.8560	3.4859	0.2456	0.7544	0.5088
8	1.2715	0.8889	2.1604	0.4115	0.5885	0.1770
20	1.0584	0.9528	2.0112	0.4738	0.5262	0.0524
100 000	1.0000	1.0000	2.0000	0.5000	0.5000	0.0000

## Linkage disequilibrium

Suppose there are *C* and *R* alleles at locus *A* and locus *B*, respectively, such that data on gametic types can be arranged into an *R×C* contingency table. Let *p*_*ij*_ be the probability of observing a haplotype with configuration ‘*ij*’. If *n* gametes are screened and the observed number having such configuration is *n*_*ij*_, the assumption of multinomial sampling leads to

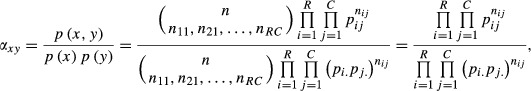

where 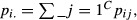 and 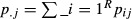 are the marginal probabilities of alleles ‘*i*’ and ‘*j*’at loci *A* and *B* respectively. Define parameters *D*_*ij*_ = *p*_*ij*_ − *p*_*i*._*p*_*j*._, measuring departure from independence (i.e. disequilibrium between alleles *i* and *j*). In a two-locus model a single *D* parameter is needed, and by using *p*_*ij*_ = *D* + *p*_*i*._*p*_*j*._, then α_*xy*_ would be the ratio between two likelihoods: the ‘unrestricted’ likelihood (indexed by parameters *D*, *p*_*i*._*, p*_*j*._) and the ‘restricted’ likelihood (indexed by the two allelic frequencies). The asymptotic connection between the standard log-likelihood ratio statistic and the *χ*^2^ metrics used for multi-locus analysis of LD by Hill (1975) and more recently by Zhao *et al*. (2005) is given, for example, in Agresti (2002).

Under this sampling scheme, the discrepancies become

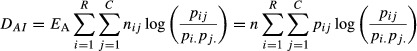

and

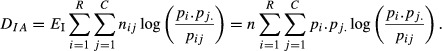


Hence

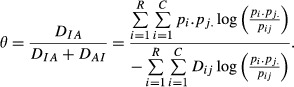


Naturally, *θ* is not defined when all *D*_*ij*_ = 0 because then the two distributions would be identical and the KL distance is 0 in that case. Note that *D*_*AI*_ is the expected value of the log-likelihood ratio, but under the assumption of association; on the other hand, when the sampling distribution of the log-likelihood statistic is considered, the reference distribution is the null model, i.e., linkage equilibrium. This illustrates an important conceptual difference, apart from the fact that *D*_*IA*_ and *D*_*AI*_ involve integration over the sampling space of allelic outcomes at the two loci in question.

To illustrate, consider a hypothetical example, patterned after data in Spiess (1989) reproduced partially by Frankham *et al*. (2010). The data pertain to three and four alleles at the major histocompatibility loci *HLA-A* and *HLA-B* respectively; the data, after a modification explained subsequently, are shown in Table 2. Frankham *et al*. (2010) presented only the data for these alleles, yet there are many more variants at these loci. Relative frequencies in Frankham *et al*. (2010) were ‘normalized’ such that the 12 joint frequencies would add up to one.

**Table 2:** Hypothetical data for major histocompatibility loci *HLA-A* and *HLA-B*, with three and four alleles respectively. The *p*_*ij*_ are haplotype frequencies; 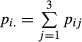 and 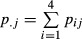 give the allelic frequencies. The hypothetical number of observed haplotypes is assumed to be 108

Locus	A1	A2	A3	Marginal
B7	*p*_11_ = 0.0270	*p*_12_ = 0.0950	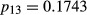	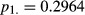
B8	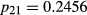	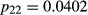	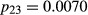	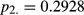
B35	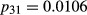	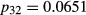	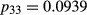	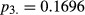
B44	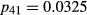	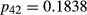	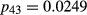	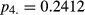
Marginal	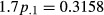	*p*_.2_ = 0.3841	*p*_.3_ = 0.3001	1.0 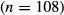

Taking haplotype frequencies *p*_*ij*_ as true values, the discrepancy of the independence model away from association is

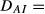


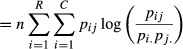


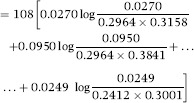


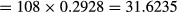


The relative contributions of each of the alleles at the *B* locus to the 0.2928 in *D*_*AI*_ above are (after rounding) 22.71%, 61.77%, 13.76% and 1.76% for *B7, B8, B35* and *B44* respectively, implying that associations with allele *B8* are responsible for most of the departure of the independence model away from association, assuming this last one is ‘true’; note that the *n*_*ij*_ do not enter into the calculations, since the frequencies are viewed as true ones. For example, the relative contribution of *B8* to *D*_*AI*_ is arrived at as follows:

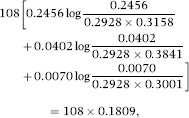

and then 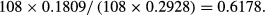 Likewise,

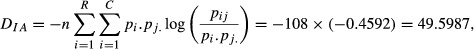

and the relative contributions of the four *B*-locus alleles to *D*_*IA*_ are 16.80%, 53.93%, 12.11% and 17.16% for *B7, B8, B35 and B44* respectively, with *B8* playing a major role again. Similar calculations can be done for the *A* locus. The KL distance is 31.6235 + 49.5987 = 81.2222, and the KL distance per haplotype scored (*N* = 108) is 81.2222/108 = 0.7521. The indexes of association are 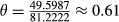
and 1 − *θ* ≈ 0.39.

In an *R×C* table of alleles, a correlation has little meaning and is not invariant with respect to how alleles are scored (e.g. 0, 1, 2) at each of the intervening loci. On the other hand, the chi-square statistics of Hill (1975) and Zhao *et al*. (2005) are also invariant and have a simple interpretation in terms of deviations expected under the null distribution. Our parameter *θ* is also score invariant, is independent with respect to the order of rows and columns, and applies to any distribution. For instance, if the simple multinomial sampling model is violated due to, e.g. over-dispersion, an appropriate *θ* could be developed under such model, whereas a ‘normalized’ *χ*^2^ would provide a crude, albeit probably useful, approximation.

The ‘relative distance’ metric takes the form

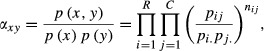

with

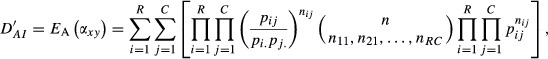

and

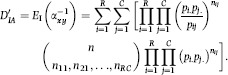


We evaluated *D′*_*AI*_*, D′*_*IA*_ and *θ*′ = *D′*_*IA*_/(*D′*_*IA*_ + *D′*_*AI*_) using Monte Carlo sampling. In the calculation of *D′*_*AI*_ one million random trials of size 108 each, with {*n*_*ij*_} varying at random, were sampled from a multinomial distribution with probabilities *p*_*ij*_; then, 
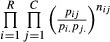
 was evaluated for each realization and averaged over trials. Similarly, one million trials of size 108 were sampled from a multinomial distribution with probabilities *p*_*i*._*p*_*j*._ and the realizations of 
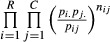
 were averaged out. The index *θ′* was estimated using the mean and median of the draws of *θ′*, yielding 0.9953 and 0.9998. The association (disequilibrium) between alleles at the *A* and *B* loci is patent, both when logarithmic (*θ*) and relative (*θ*′) distances are employed to measure it.

## Systems of multivariate beta processes

### Bivariate beta distributions

A common finding in studies of population differentiation using Wright's *F*-statistics (Wright 1931; Cockerham 1969) with single nucleotide polymorphisms (SNPs) is that estimates of allelic frequencies within a bin of adjacent SNPs are correlated (e.g. Akey *et al*. 2002; Weir *et al*. 2005; Akey 2009). This is typically due to stochastic dependence between alleles at linked loci (producing LD), but evolutionary processes causing dependence among the true frequencies themselves have been suggested; see Ohta (1982) for a population genetics model based on this concept. For example, a correlation between alleles at randomly drawn pairs of loci arises if alleles are conditionally (given the allelic frequencies) independent but with allelic frequencies varying at random according to a beta distribution.

Suppose that pairs of loci are sampled at random from some conceptual population of equi-correlated loci and that all pairs of ‘true’ frequencies define a probability distribution. Wright (1937) found that a beta distribution arose from a diffusion equation used to study changes in allele frequencies in finite populations, so the beta process is well grounded in population genetics. Beta distributions have been used in studies of population differentiation as mixing processes to create randomness of allelic frequencies, leading to beta-binomial likelihoods or to unrecognized posterior distributions that must be resolved using sampling procedures, e.g., Holsinger (1999), Balding (2003), Beaumont & Balding (2004) and Gianola *et al*. (2010).

Similarly, it would seem sensible to assume that allelic frequencies of pairs of loci within the hypothetical population have an association that can be modelled with bivariate beta distributions to represent random variation of true, albeit unknown, allelic frequencies. For example, suppose that there are *S* half-sib families that can be viewed as drawn as random from some population and that we are interested in modeling association between pairs of allelic frequencies stemming from such sampling scheme. Here, distributions proposed by Sarmanov (1966) and Olkin & Liu (2003) are discussed and generalized as candidate processes that could be used to model this type of association.

### Olkin-Liu bivariate beta distribution

First, we review how a specific bivariate beta distribution arises. As in Olkin & Liu (2003), let *U, V* and *W* be random variables following independent standard Gamma distributions with parameters *a, b* and *c* respectively. By construction, 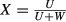 and 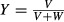 possess the beta distributions *X*: *Beta*(*a,c*) and *Y*: *Beta*(*b,c*) respectively. Clearly, *X* and *Y* are positively correlated (through *W*), with a strength that depends on the values of *a, b, c*. Olkin & Liu (2003) show that *X* and *Y* have a bivariate beta distribution with density function

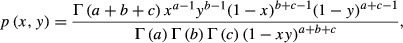
(14)

**Figure 4:**
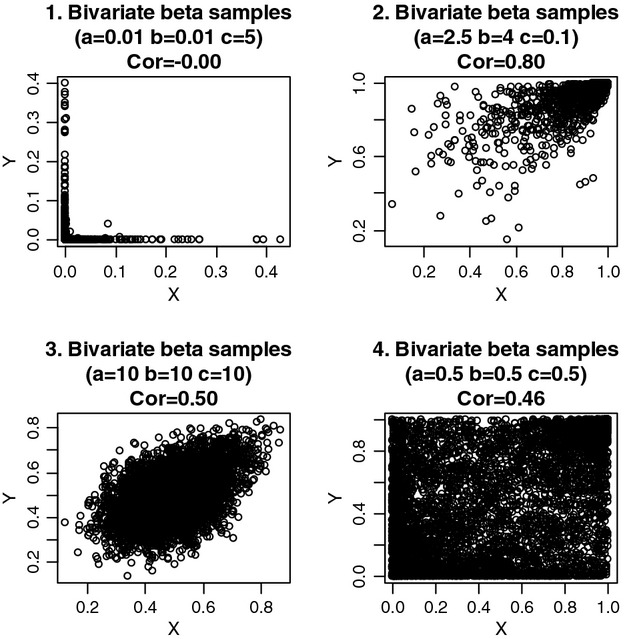
Scatter diagrams of 5000 samples from each of four Olkin-Liu bivariate beta distributions. Plot 1 illustrates a strong association with essentially no correlation. Plot 2 (‘meteorite’) depicts a limitation of the correlation as a parameter for describing association. Plot 3 suggests association clearly. Plot 4 shows a bivariate distribution that is not trivial: the true correlation (0.46) arises primarily due to weaker association in the ‘middle’ of the bivariate sampling space.

Where 0 < *X* < 1 and 0 < *Y* < 1. Moments *E*(*X*^*k*^*Y*^l^) cannot be written in closed form but can be approximated numerically or using sampling methods. Large *c* and small *a, b* produce correlations close to 0, whereas large *a, b* or small *c* produce correlations close to 1 (Olkin & Liu 2003). For example, a correlation equal to 0.002 is obtained for *a* = *b* = 0.01 and *c* = 5, whereas the correlation is 0.91 for *a* = 2.5, *b* = 4 and *c* = 0.1. Figure 4 displays scatter plots of 5000 samples obtained from each of four bivariate beta distributions. Plot 1 represents a distribution in which the correlation is very low, and yet, there is considerable association between pairs of values near 0, illustrating inadequacy of correlation to reveal association. In plot 2 (resembling a ‘meteorite’) clustering takes place primarily at large values of *X* and *Y*. The two bottom plots depict bivariate beta distributions with similar correlations but with a completely different pattern of association. Clearly, correlation often fails as measure of statistical association.

In this bivariate beta model the density ratio takes the form

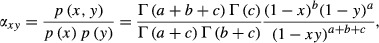
(15)

Where *p*(*x*) and *p*(y) are the beta densities of *X* and *Y*: *Beta*(*b,c*). Then

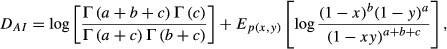


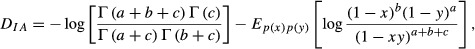

so that

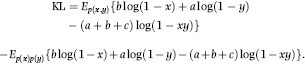
(16)

The corresponding *D′*_*AI*_*, D′*_*IA*_ and KL*′* can be calculated by taking expectations of density ratios as opposed to expectations of their logarithms.

Indexes of association based on *D′*_*AI*_*, D′*_*IA*_ and KL*′* were calculated for the following combinations of parameters, where *ρ* denotes the expected correlation: 1) *a =* 0.01, *b* = 0.01, *c* = 5 (*ρ* = 0.002); 2) *a =* 1, *b* = 0.5, *c* = 0.6 (*ρ* = 0.251), 3) *a* = 1, *b* = 1, *c* = 0.9 (*ρ* = 0.496) and 4) *a* = 2, *b* = 3, *c* = 0.4 (*ρ* = 0.750). Expectations under the independence model were approximated by drawing 1 million random numbers from independent *X ∼ Beta*(*a,c*) and *Y ∼ Beta*(*b,c*) distributions, and then averaging the evaluations of the appropriate expressions over the draws. In the association model, 1 million random triplets (*U,V,W*) were drawn from independent standard gamma variables with parameters *a, b* and *c*, to then form bivariate realizations as 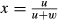 and 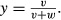 Table 3 gives the results, and all quantities (save for the true *ρ*_*XY*_) are Monte Carlo estimates. The indexes of association *θ′* and 1 − *θ′* departed from 0.5 and approached 0 and 1, respectively, as the correlation in the bivariate beta distribution became stronger. Likewise, *γ′* and *γ′′* drifted away from 0, approaching |1|. In settings (3) and (4), *θ′* and 1 − *θ′* suggest a stronger association than what would be indicated by *ρ*.

**Table 3:** Departures of Olkin-Liu bivariate beta distributions from independence as measured by *D′*_*AI*_, *D′*_*IA*_ and by the indexes of association *θ′* and 1 − *θ′* (when 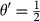 independence holds), for four combinations of parameters *a, b* and *c*. 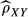 is the Monte Carlo estimate (1 million samples) of the true correlation *ρ*_*XY*_. Means of variables *X* and *Y* are estimates from samples from association (*A*) or independence (*I*)models. The *D′*_*AI*_ and *D′*_*IA*_ parameters are estimates under the appropriate asumptions; KL*′* = *D′*_*AI*_ + *D′*_*IA*_; *θ′* = *D′*_*IA*_/(*D′*_*AI*_ + *D′*_*IA*_); *γ′* = 2*θ′* − *1*; *γ′′* = 1 − 2*q′*

Item	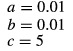	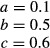	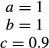	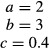
*ρ*_*XY*_	0.002	0.2510	0.4960	0.7500
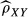	0.002	0.2499	0.4951	0.7304
ave. (X)	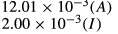	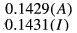	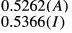	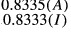
ave. 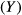	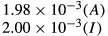	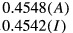	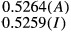	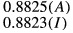
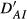	0.9999	0.8709	0.4292	0.0745
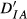	0.9999	0.8009	0.0779	1.944 × 10^−5^
KL^′^	1.9999	1.5781	0.5071	0.0745
*θ*^′^	0.5000	0.4481	0.1537	0.0003
1 − *θ*^′^	0.5000	0.5519	0.8463	0.9997
*γ*^′^	0.0000	−0.1037	−0.6926	−0.9995
*γ*^′′^	0.0000	0.1037	0.6926	0.9995

### Olkin-Liu bivariate beta-binomial distribution

In studies of LD, focus is typically on correlation between alleles rather than between frequencies. We discuss how a correlation between alleles can arise when the association stems from frequencies, as could happen when the population consists of clusters of individuals resulting from some family structure. Consider bi-allelic locus *l* with allelic frequencies Pr(*A*_*l*_) = *p*_*l*_ and Pr(*a*_*l*_) = 1 − *p*_*l*_; *A*_*l*_ and *a*_*l*_ denote the two alleles at locus *l*. Suppose that a sample of *N* individuals is scored, and that the observed number of copies of *A*_*l*_ and *a*_*l*_ are 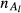 and 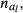 respectively, with 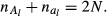 Given *p*_*l*_, the distribution of 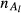 is *Binomial*(2*N*,*p*_*l*_). Now, let the allelic frequency *p*_*l*_ vary at random among clusters (e.g. sub-populations) according to a beta distribution with parameters (*a,c*). Then, the marginal distribution of the observed number of alleles is beta-binomial (e.g., Casella & George 1992; Sorensen & Gianola 2002), that is, the probability of observing 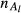 copies among the 2*N* alleles scored is

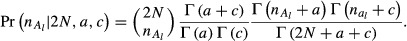
(17)

Consider now a pair of loci, and let the observed number of copies of the four alleles be represented as 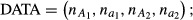 haplotype frequencies are not relevant in the model that follows. If, given the allelic frequencies **p** = {*p*_*l*_}, the number of copies of *A*_1_ and *A*_2_ are independently distributed, i.e., the two loci are in linkage equilibrium, the distribution of the observed number of alleles is

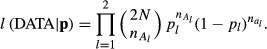
(18)

Suppose now that this pair is a realization from a stochastic process where loci are sampled over a conceptual population of pairs, e.g. the two loci are drawn at random from a physical ‘bin’ of a certain length in kilobases. This process generates a covariance structure between allelic frequencies of pairs of loci within the ‘bin’. The alleles would be in LD, marginally, even though being in conditional (given the allelic frequencies) equilibrium. If the unobserved, ‘true’, allelic frequencies follow some bivariate distribution with density *g*(*p*_1_,*p*_2_|**θ**), where **θ** denotes its parameters, the density of the marginal distribution of the observed number of allelic counts under this association model would be

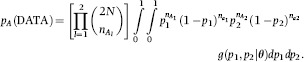
(19)

If a bivariate Olkin-Liu beta distribution with parameters **θ **= (*a,b,c*) is assumed, the joint density of allelic frequencies *p*_1_ and *p*_2_ as in (14). Letting

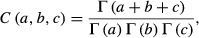

the marginal distribution (19) takes the form

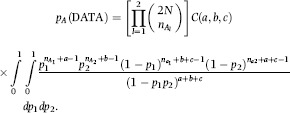
(20)

This integral cannot be written in closed form, but it can be evaluated using Monte Carlo methods such as importance sampling (e.g. Sorensen & Gianola 2002). One possible scheme is outlined in the Appendix.

Under this distribution

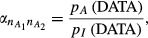


Where *p*_*I*_(DATA) is the distribution under independence between observed allele numbers, and given by the product of two beta-binomial distributions, that is

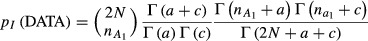


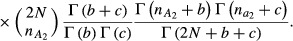
(21)

Then

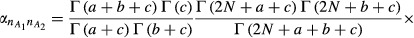


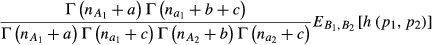
(22)
where 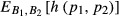 is defined in the Appendix. Preceding the expectation, the first term involves the parameters of the bivariate beta distribution, the second involves sample size (*N*) as well, and the third one depends on observed allelic counts.

The logarithmic and relative distances between distributions are calculated as

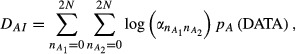


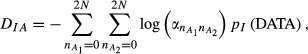


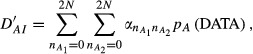

and

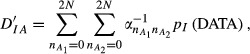


With *θ* and *θ′* calculated as before. Calculations proceed on an entirely numerical basis.

A generalization of the beta process to a situation with more than two loci is in Olkin & Liu (2003). For *K* loci the joint density of the allelic frequencies takes the form

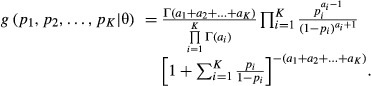


The marginal distribution of allelic counts is obtained by mixing a multinomial distribution over the multivariate beta density above, but results are not available in closed form, so a numerical solution is needed again.

### Lee-Sarmanov bivariate beta distribution

Lee (1996) proposed a different bivariate beta distribution following Sarmanov (1966); see Danaher & Hardie (2005). A Lee-Sarmanov bivariate beta distribution for allelic frequencies *p*_1_ and *p*_2_ has density

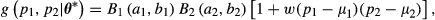
(23)

Where *B*_1_(.) and *B*_2_(.) are beta densities as before and *w* is a parameter (*w* = 0 produces independence); θ* = (*a*_1_,*b*_1_,*a*_2_,*b*_2_,*w*). Above, 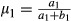 and 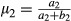 are the expected values of *p*_1_ and *p*_2_ respectively. The marginal densities of *p*_1_ and *p*_2_ under this model are *B*_1_(.) and *B*_2_(.). Further,

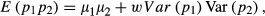


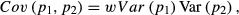

so that

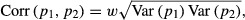

with

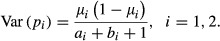


In the Lee-Sarmanov bivariate beta distribution, a null correlation implies independence. Under this model the density ratio becomes

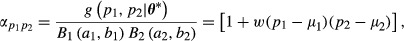

so

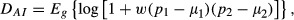

where *E*_*g*_ denotes expectation under association and

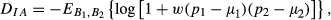

is the corresponding expectation under independence of the distributions. Further

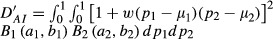


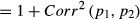
(24)
is in closed form, while

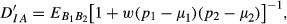
(25)
can be estimated using Monte Carlo sampling, assuming this expectation exists. Hence

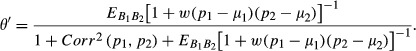


The index *θ′* was evaluated for three sets of values of the *a,b* parameters for each of the two intervening beta distributions while setting the correlation at 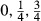 or 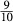. Metric *D′*_*AI*_ in (24) follows directly from the correlation, while *D′*_*IA*_ was estimated using 1 million random samples from each of the two beta distributions. For each set of *a,b* parameters *w* was calibrated via

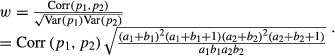


**Figure 5:**
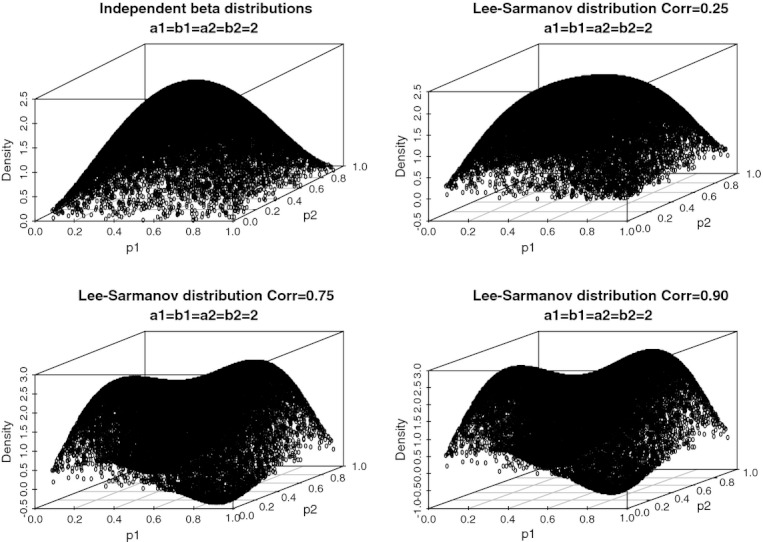
Plots of the densities of four Lee-Sarmanov bivariate beta distributions having the same Beta (2,2) marginal distributions but differing in the strength of association: as the correlation increases multi-modality emerges.

Note that *D′*_*AI*_ is solely a function of the correlation, so it was the same for the three pairs of beta distributions examined. Using (25), *D′*_*IA*_ was estimated by averaging 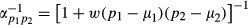 over draws. Because 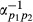
is a ratio between density functions, negative realizations were not used when forming the Monte Carlo estimate (this produces an upward bias but a more precise estimate). As shown in Table 4, the KL distance between the bivariate beta distribution and the independence process increased monotonically with the strength of the correlation. The indexes of association *θ′* and 1 − *θ′* also drifted away monotonically from 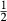 only in setting 2. For instance, in setting 1 *θ′* increased from 0.50 to 0.66 as the correlation increased from 0 to 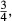 but decreased to 0.60 when the correlation was 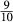. The shape of the bivariate beta distribution was examined for this setting by plotting the density at 20 000 random points drawn from each of the marginal *Beta*(2,2) distributions. This is shown in Fig. 5: the Lee-Sarmanov density ‘evolves’ towards a more complex topography as correlation increases. When correlation grows from 0 to 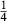, the shape of the joint distribution is not too different from that under independence, as also suggested by KL and *θ′*. However, when the correlation increases by a factor of 3 from 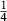 to 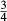, the KL distance grows only about two times, while *θ′* and 1 − *θ′* move away from 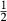 at an even slower rate; this is expected because *θ* has an upper bound at 1, whereas KL takes any positive value in the real line. The bimodal shape of the density also suggests that more intensive sampling is needed to obtain reliable estimates of the indexes of association.

**Table 4:** Departures of Lee-Sarmanov bivariate beta distributions with varying correlation from independence, as measured by *D′*_*AI*_, *D′*_*IA*_ and by indexes of association *q′* and 1 − *q′* (when 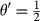 independence holds). The four correlations levels depend on the value of parameter *w* of the bivariate distribution, and on parameters *a,b* of the two ‘parental’ beta distributions. *D′*_*AI*_ has closed form and *D′*_*IA*_ was estimated using 1 million samples from the beta distributions. KL*′* = *D′*_*AI*_ + *D′*_*AI*_; *q′* = *D′*_*IA*_/(*D′*_*AI*_ + *D′*_*IA*_)

Setting/Item	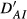	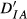 (% negative)	KL^′^	*θ*^′^	1 − *θ*^′^
(1) *a*_1_ = *b*_1_ = *a*_2_ = *b*_2_ = 2					
Correlation (*w*)					
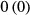	1	1	2	0.5000	0.5000
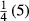	1.0625	1.2681 (0.0)	2.3307	0.5441	0.4559
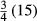	1.5625	3.0985 (8.5)	4.6610	0.6648	0.3352
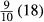	1.8073	2.7369 (11.2)	4.5468	0.6019	0.3981
(2) *a*_1_ = 10; *b*_1_ = 90; *a*_2_ = 90; *b*_2_ = 10					
Correlation (*w*)					
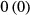	1	1	2	0.5000	0.5000
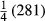	1.0625	1.1466 (0.4)	2.2091	0.5190	0.4810
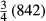	1.5625	2.6824 (6.3)	4.2449	0.6319	0.3681
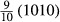	1.8073	4.4937 (8.7)	6.3037	0.7128	0.2871
(3) *a*_1_ = .25; *b*_1_ = .75; *a*_2_ = .75; *b*_2_ = .25					
Correlation (*w*)					
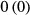	1	1	2	0.5000	0.5000
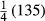	1.0625	1.3766 (0.9)	2.4391	0.5644	0.4356
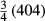	1.5625	1.5094 (4.9)	3.0719	0.4914	0.5086
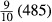	1.8073	1.5876 (5.6)	3.3976	0.4673	0.5327

As shown in Danaher & Hardie (2005), the marginal distribution of the observed allelic counts 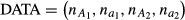 is obtained by mixing (18) over the Lee-Sarmanov density (23), leading to

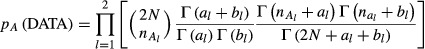


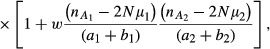
(26)

Using the fact that under independence the joint distribution is product beta-binomial as in (21), it turns out that

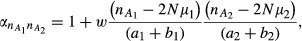

so that closed forms are available for

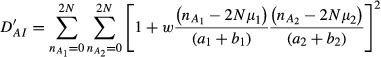


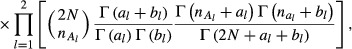

and

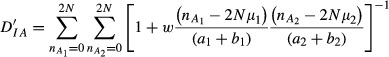


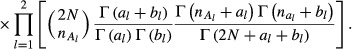


The correlation between number of copies of the *A* alleles at the two loci is (Danaher & Hardie 2005)

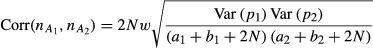


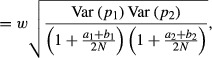

so that for large *N*

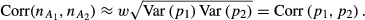


A multivariate generalization of (26) is direct but appears that it has not been reported elsewhere. The joint density of the allelic frequencies at *L* loci takes the form

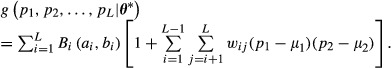
(27)

It is straightforward to verify that this density integrates to 1, and that all marginal distributions are beta. Now, let

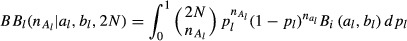


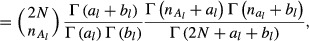

denote the beta-binomial distribution of the observed number of copies of allele *A*_*l*_, out of 2*N* copies. The marginal distribution of allelic counts over all loci can be shown to be, after algebra

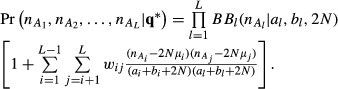
(28)

We propose (for obvious reasons) to name (27) and (28) as multivariate beta GMS-Sarmanov and multivariate beta-binomial GMS-Sarmanov distributions, respectively.

## Discussion

The problem addressed in this study was that of measuring departures of the joint distribution of genetic variables from independence. New measures of association based on notions of statistical distance were proposed and evaluated under several scenarios, spanning from multivariate Gaussian distributions modeling, say, pleiotropic effects, to systems of beta distributions describing association between allelic frequencies. Two hereto seemingly unreported probability distributions were derived, and termed GMS-Sarmanov multivariate beta and GMS-Sarmanov multivariate beta-binomial distributions. The standard LD problem was also dealt with to illustrate the generality of the approaches proposed.

Linkage disequilibrium analysis has been the subject of an enormous amount of research, re-energized with the availability of massive molecular marker and sequence data. For example, in the context of coalescent theory, an important issue is the decoupling of ancestries of sites at different regions of the chromosome, and this is done by studying association between alleles at different loci (Wakeley 2009). Most of the standard measures of LD employed are pairwise statistics such as correlations (e.g. Hill & Robertson 1968; Hill 1974; Hedrick 1987; Lewontin 1988; Morton *et al*. 2001; Pritchard & Przeworski 2001; McVean 2007), because of ease of calculation and, perhaps, ease of interpretation. However, as pointed out by Lewontin (1988), although a correlation is a meaningful parameter (e.g., in terms of the amount of variability of *Y* explained when *X* is observed) in a bivariate normal distribution, it is arguably less so when applied to discrete data, a problem that is well known in quantitative genetic analysis of discrete phenotypes (Dempster & Lerner 1950; Gianola 1982). Further, pairwise measures do not characterize association well if a given genetic system involves many correlated random variables, as in multi-locus measures of LD. Actually, such measures have been reported much less often, e.g. Weir (1996) gives formulae for up to four loci, and Sabatti and Risch (2002) give a measure based on excess of heterozygosity or of homozygosity.

Nothnagel *et al*. (2002) presented an entropy-based index of LD that is related to our approach, but that differs in some respects. These authors calculate the entropy of the allelic distributions under linkage equilibrium and under disequilibrium, and express the difference in entropy as a fraction of the equilibrium entropy. This produces a normalized entropy that takes values between 0 and 1 (0 indicating no association between alleles at different loci). A formal objection is that, while entropy measures uncertainty in a distribution, its relationship to statistical distance between distributions (Kullback 1968) requires more elaboration. Their method is for bi-allelic loci only, and entropy does not always behave well in continuous distributions (Bernardo and Smith, 1994; Sorensen & Gianola 2002), whereas relative entropy measures such as the KL distance are well defined.

Closer to the spirit of our procedures, Liu & Lin (2005) suggested a measure of LD based on a relative KL discrepancy, but it differs from the ones we propose. This is mainly because they use only one of the two components (termed *D*_*AI*_ in our paper) of the invariant KL distance, and express it relative to the maximum value it can take. Using their procedures with *D*_*IA*_ would produce a different value of association, and maybe a different qualitative picture might emerge from analysis of genetic data. However, their ideas can be embedded in our approach, and expressing association relative to a maximum distance is a well taken point. It also turns out that these results can also be adapted to the continuous domain, with some care. To illustrate this, it suffices to consider two random vectors, **x** and **y**, as generalization to a higher-dimensional system is straightforward. Let *H*(**x**), *H*(**y**), and *H*(**x,y**) be the entropies (non-negative, by construction, although pathological examples may arise in continuous distributions) of the marginal distributions of **x**,** y**, and **x,y** respectively, with

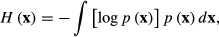


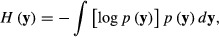

and

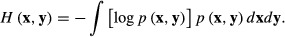


The discrepancy away from the association model is

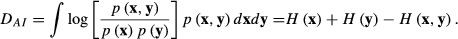
(29)

Note that

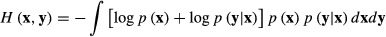


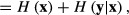


Where *H*(**y**|**x**) is the entropy of the conditional distribution of **y** given **x**. This implies that *H*(**x**,** y**) ≧ *H*(**x**), where **x** can also be any partition of **x**, e.g. its *i*th coordinate *x*_*i*_, say, and similarly *H*(**x**,** y**) ≧ *H*(**y**). Thus, *H*(**x**,** y**) ≧ max_*i*_ *H*(*x*_*i*_). Using this in (29)

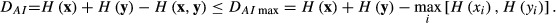


More generally, for a vector **z** with *n* elements, the KL discrepancy away from the independence model is

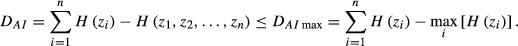
(30)

Likewise

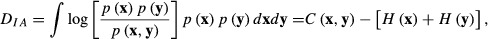

where

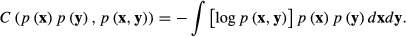

is the cross-entropy between distributions. From the expressions for *D*_*AI*_ above, *H*(**x**) + *H*(**y**) ≧ max_*i*_[*H*(*x*_*i*_),*H*(*y*_*i*_)], so that

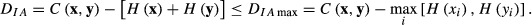


This leads to additional indexes of association, such as

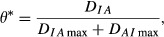
(31)
and

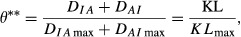
(32)
both taking values between 0 and 1. These indexes were evaluated for a bivariate normal distribution with correlation 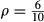 and marginal distributions *x*: *N*_1_(0,1) and *y*: *N*_2_(0,1). One obtains

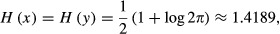


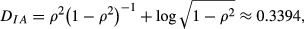


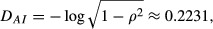


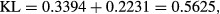


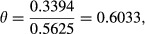


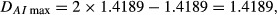


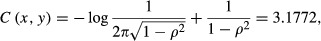


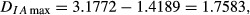


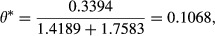

and

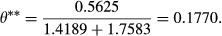


The four indexes, ρ, θ, θ** and q** produce a different value of the strength of association between random variables in a distribution. This suggests that probably there is no such thing as a universal measure, although it is clear that the coefficient of correlation lacks generality.

In a nutshell, this paper presents measures of association for systems of genetic variables that go beyond standard two-dimensional statistics. The procedures apply to either continuous or discrete data, and typically require numerical implementation, because many of the expressions are not available in closed form, depending on the distribution assumed. Our procedures (like any other method) require knowledge of the parameters of the distributions under independence or association, and estimation of such parameters is not the objective of this paper. Today, computer-intensive approaches for parameter inference, such as Bayesian Markov chain Monte Carlo (Sorensen & Gianola 2002; Gelman *et al*. 2004) or approximate Bayesian computation (Beaumont *et al*. 2002), can be implemented effectively in today′s high throughput systems (Wu *et al*. 2011).

Finally, since this volume is in honor of the contributions of Professor Moshe Soller, a relationship between this paper and his work should be established. As noted at the onset of this document, among many other accomplishments, he pioneered marker-assisted selection in animal and plants via exploitation of linkage and LD relationships between markers and unknown quantitative trait loci. Examples of his papers in this area include Soller & Genizi (1978) and Lipkin *et al*. (2009), and these connect with some of the developments presented here. We look forward to many more scientific accomplishments by Moshe!

## Appendix

### Bivariate and univariate t-distributions

The density of a *K*-variate random vector following a multivariate *t*-distribution is

(33)

where  **Σ** is the scale matrix, *υ* > 0 is the degrees of freedom parameter and  is the Gamma function; the covariance matrix of this distribution is  The specification *k* = 2, **μ** = **0** and **Σ** = **I**_2_, yields a bivariate *t*-distribution with null mean and covariance matrix   the two marginal distributions are *univariate*-*t*, each having a null mean, variance  and *ν* degrees of freedom. Although uncorrelated, these two random variables are statistically dependent because

The draws from the bivariate *t*-distribution needed for evaluating  and  are obtained as

Under independence, the sampling from 2 univariate *t*-distributions is done as

Above, the *z*^’^*s* are independent draws from   is a draw from a central chi-square distribution on *ν* degrees of freedom, and  and  are two independent draws from the same distribution.

### Olkin-Liu bivariate beta-binomial distribution: sampling scheme

When allelic frequencies are independent, their joint density is the product of the densities of the beta random variables  and  However, this does not provide a suitable importance sampling distribution, because dependency would not be recognized at all, with the sample space not visited appropriately. A form of introducing dependency is via an importance sampling density with two dependent beta distributions, such as

(34)

Distribution  depends on *p*_1_, and has expectation

which would be equal to the mean value under independence only if *p*_1_ = 0.5. The double integral in (20) can be represented as

where

and  denotes expectation with respect to the joint distribution with density  Using this in (20), the marginal distribution of the data under the association model is

(35)

Expression (35) is a seemingly unreported discrete probability distribution which we term ‘Olkin-Liu bivariate beta-binomial distribution’.
